# Non-Invasive Label-free Analysis Pipeline for In Situ Characterization of Differentiation in 3D Brain Organoid Models

**DOI:** 10.21203/rs.3.rs-4049577/v1

**Published:** 2024-04-01

**Authors:** Caroline Filan, Seleipiri Charles, Paloma Casteleiro Costa, Weibo Niu, Brian F. Cheng, Zhexing Wen, Hang Lu, Francisco E. Robles

**Affiliations:** 1 Georgia Institute of Technology, George W. Woodruff School of Mechanical Engineering, Atlanta, GA, 30318, USA; 2 Georgia Institute of Technology, Interdisciplinary Program in Bioengineering, Atlanta, GA, 30332, USA; 3 Georgia Institute of Technology, School of Electrical & Computer Engineering, Atlanta, GA, 30332, USA; 4 Emory University School of Medicine, Department of Psychiatry and Behavioral Sciences, Atlanta, Georgia 30322, USA; 5 Georgia Institute of Technology and Emory University, Wallace H. Coulter Department of Biomedical Engineering, Atlanta, GA, 30318, USA; 6 Emory University School of Medicine, Departments of Cell Biology and Neurology, Atlanta, Georgia, 30322, USA; 7 Georgia Institute of Technology, School of Chemical & Biomolecular Engineering, Atlanta, Georgia 30332, USA

## Abstract

Brain organoids provide a unique opportunity to model organ development in a system similar to human organogenesis *in vivo*. Brain organoids thus hold great promise for drug screening and disease modeling. Conventional approaches to organoid characterization predominantly rely on molecular analysis methods, which are expensive, time-consuming, labor-intensive, and involve the destruction of the valuable 3D architecture of the organoids. This reliance on end-point assays makes it challenging to assess cellular and subcellular events occurring during organoid development in their 3D context. As a result, the long developmental processes are not monitored nor assessed. The ability to perform non-invasive assays is critical for longitudinally assessing features of organoid development during culture. In this paper, we demonstrate a label-free high-content imaging approach for observing changes in organoid morphology and structural changes occurring at the cellular and subcellular level. Enabled by microfluidic-based culture of 3D cell systems and a novel 3D quantitative phase imaging method, we demonstrate the ability to perform non-destructive high-resolution imaging of the organoid. The highlighted results demonstrated in this paper provide a new approach to performing live, non-destructive monitoring of organoid systems during culture.

## Introduction

Developing treatments for brain diseases requires understanding the human brain’s anatomy, connectivity, and function, necessitating suitable pre-clinical models. Three-dimensional (3D) stem cell cultures (termed organoids) have been developed from human induced-pluripotent stem cells (iPSCs) to address the lack of robust pre-clinical models that accurately recapitulate human brain development.^1–5^ Although human organoids offer a unique opportunity for modeling organ development, a major challenge is the inability to perform detailed non-invasive *in situ* imaging with access to cellular and subcellular structures. To obtain access to the cellular/subcellular structure (as well as molecular composition), imaging methods have been employed using fluorescence-based imaging modalities,^6,7^ light-sheet imaging,^8^ and immunohistochemistry.^1,5,9–18^ These platforms, however, primarily rely on end-point assays, which are destructive, time-consuming, and labor-intensive. This reliance on end-point assays is partially due to the opaqueness of the millimeter-sized organoids, which makes it challenging to assess cellular and sub-cellular events occurring during organoid development in their 3D context. As a result, only some of the long (often several months) developmental processes are monitored/assessed.

To gain insight into the overall health of the organoid during culture in a non-invasive manner, brightfield imaging is commonly employed. This technology is simple, ubiquitously available, and provides meso-scale structures of the organoid such as volume and circularity, estimated from 2D projection images;^9,19–21^ however, it lacks the cellular and sub-cellular detail. More recently, optical coherence tomography (OCT) has also been applied to monitor organoids non-invasively,^22,23^ but OCT is generally unable to resolve cellular or subcellular structures.

Here, we apply an emerging imaging technology called quantitative oblique back-illumination microscopy (qOBM) to non-destructively image cerebral organoids *in situ*.^24–26^ qOBM enables 3D quantitative phase imaging (QPI) with epi-illumination; and like QPI^27–31^ qOBM provides clear, quantitative contrast of cellular/subcellular structures, but with the significant and unique advantage that qOBM can do so in 3D, in thick scattering samples with epi-illumination.^32–34^ With inherent refractive index (RI) contrast, qOBM yields unprecedented access to subcellular structural detail of organoids in situ without exogenous labels and is an optimal method for imaging brain organoids and monitoring their development during culture.

In this paper, we develop a non-invasive pipeline for the live, non-destructive, longitudinal monitoring of organoids using microfluidic technology, qOBM, and brightfield microscopy. We leverage microfabricated technologies which offer increased customizability of device configurations and have demonstrated potential in increasing cellular diversity, reducing necrotic core formation, and disease modeling in organoid models.^9,21,35,36^ First we determine microfluidic device design parameters for long-term culture with proper nutrient exchange while permitting monitoring with brightfield imaging and qOBM. We then apply this integrated system to image healthy and disease organoid models. We demonstrate high-content imaging to identify cellular phenotypes and longitudinal imaging to observe subtle morphological and potential metabolic changes. Our combined brightfield-qOBM-microfluidics system has the potential to non-invasively differentiates between disease and non-disease states in organoid models, providing insights for downstream molecular analysis. Together, the proposed pipeline comprises a new approach for live, non-destructive monitoring of organoid systems.

## 1 Results

### 1.1 Non-destructive imaging pipeline of brain organoids

**Figure 1.**
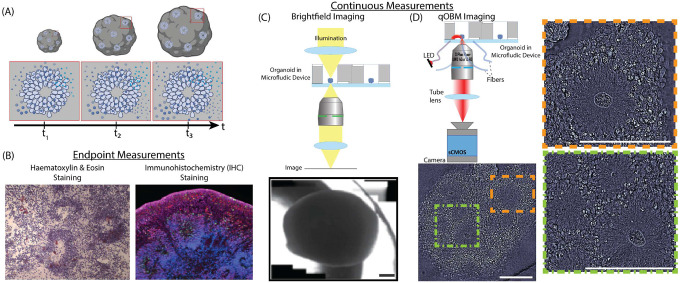
Conventional and Proposed Organoid Analysis Pipeline: (A) schematic of organoid growth over time, including increase in size–which can be monitored with brightfield–and development of neuro-progenitor structures with cells eminating from the center–which can be observed with qOBM. (B) conventional endpoint analyses including Haematoxylin & Eosin (H&E) staining and immunohistochemistry (IHC) staining. (C) schematic of brightfield imaging of the organoids in the custom microfluidic devices with a representative image. (D) schematic of the qOBM imaging system used for the organoids in the microfluidic device. Bottom contains a 20X image. Right insets contain 40X images of the indicated regions in the 20X image. Scale bars are 200 *μ*m.

Our combined imaging approach for label-free imaging of 3D organoids involved performing brightfield imaging and qOBM imaging in an optimized microfluidic platform. Utilizing both imaging modalities enables high-content non-destructive analysis of organoids which can then be used to guide further downstream analysis (Figure 1).

The qOBM system consists of a conventional brightfield microscope with a modified illumination module.^24–26,34^ Rather than the classic transmission-based illumination used in brightfield microscopy, qOBM illuminates samples using four LED light sources (720 nm) deployed through optical multimode fibers arranged around the objective, 90° from each other, as seen in Figure 1D. With this configuration, the light effectively illuminates the focal imaging plane at a net oblique angle which provides phase contrast. Using a deconvolution algorithm, we obtain quantitative phase contrast images which encode the refractive index properties of the samples.^24–26, 34^ The images provide 3D cellular and sub-cellular contrast up to 190 μm into the organoid and enables us to track differences in organoid growth over time in a non-invasive manner using a simple and low cost system.

The organoids in the microfluidic device are first imaged using brightfield microscopy to capture their gross morphology, as seen in Figure 1C. Next, they are transferred to the qOBM setup to characterize organoid cell morphology, as seen in Figure 1D.

### 1.2 Microfluidic platform for non-invasive imaging of organoids

The microfluidic device can be used as a culturing and imaging platform or just an imaging platform depending on the application. When utilized as an imaging and culture platform, the device geometry has to be jointly optimized to maintain the overall organoid health and to enable live imaging of organoids via qOBM and brightfield imaging (Table S1). First, depths of culture chamber and channels are considered. The microfluidic device is fabricated by bonding two PDMS layers together. These PDMS layers are bonded to a glass slide to facilitate high-content imaging. The bottom PDMS layer determines the height of the culture chamber, while the top PDMS layer provides the additional channel or flow path needed for creating crossflow or convective flow in the device wells. To ensure proper growth of organoids in the microfluidic device, we evaluated two criteria: the shear stress experienced by the organoids and the robustness of the fabrication and operation of the devices. We first evaluated the effect of the device configuration on the shear stress experienced by the organoids in the device wells since shear stress could affect cell behavior and phenotypic expression.^37^ The shear stress was calculated using the equation below:^38^

(1) $$T=\frac{5\mu Q}{wh^{2}}$$

where *T* is fluid shear stress, μ is viscosity of water, *Q* is the volumetric flow rate, *h* is the main channel height, and w is the main channel width. Since the shear stresses calculated for the different device configurations (range: 0.004–0.03) were less than 1 cm^−2^ (upper bound determined from^38^), we assumed that shear rate did not have a considerable effect on organoids grown in our system and would not change drastically with the device configurations that were tested.

**Figure 2.**
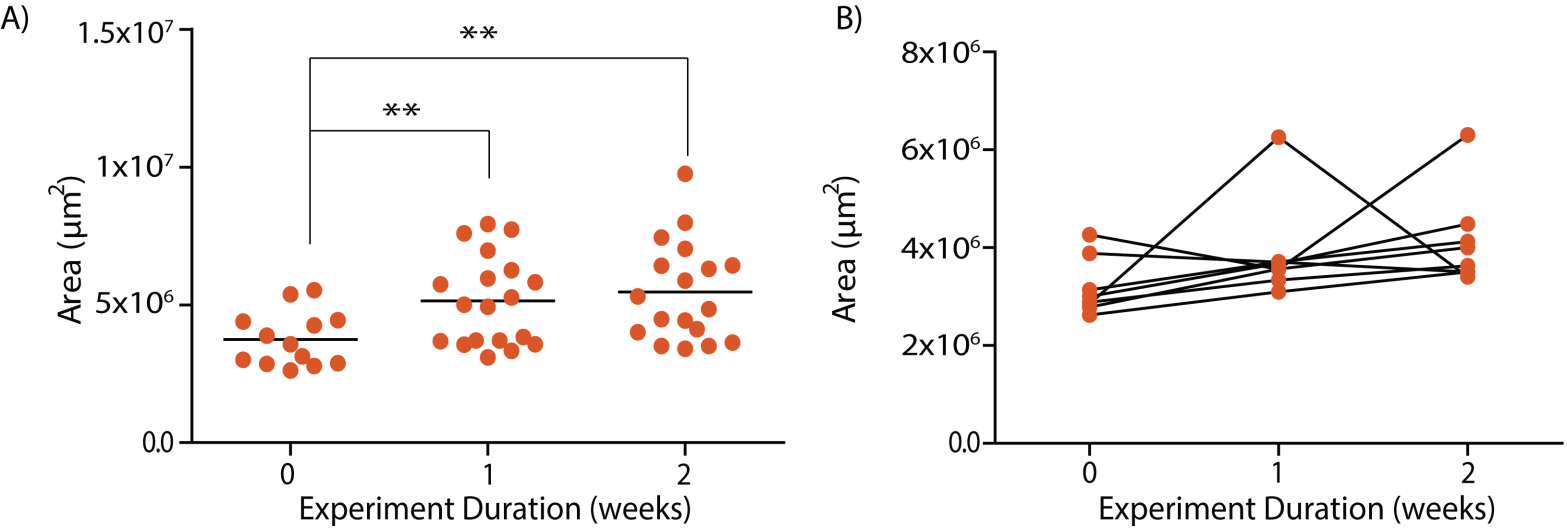
Characterization of organoid growth via brightfield imaging. (A) Characterization of the area of all organoids cultured in the microfluidic platform with device height of 7.5mm. Significance was calculated using a two-tailed unpaired t-test with Welch-correction for two groups. (B) Subset of data in (A) showing longitudinal changes in area of organoids grown in the microfluidic platform. N=6 organoids from both the control and experimental groups.

The second criterion for optimizing the device configurations involved evaluating the robustness of the device fabrication and operating processes. For example, a 1mm device height would not operate robustly under convective flow due to the instability of the inlet and outlet ports of the device, which could lead to leaks and subsequent cell death due to contamination or non-robust media exchange. Additionally, a device with a low volume would result in faster nutrient depletion during imaging sessions as the devices are disconnected from the pump set up for imaging. Empirically, we determined that we could reduce the well height (bottom PDMS layer) to ~2.5mm while maintaining the top layer’s height at ~4mm to stabilize the inlet and outlet ports of the device. Finally, we designed the well diameter to be 8mm to compensate for the loss of volume due to the reduced height (Figure S1A). Based on this optimization, we proceeded with devices ~7.5mm in total height. Next, we evaluated how well organoids could grow in the modified microfluidic device. We used the organoid size (as indicated by area) obtained from brightfield imaging to approximate organoid growth (Figure S1B). We observed that the modified platform could maintain organoids in culture for at least two weeks, as indicated by the increase in the organoid area during this time (Figure 2). As we show in Figure 1, this device geometry is also well suited for qOBM and brightfield imaging.

### 1.3 Imaging human brain organoids

To demonstrate the utility of our imaging approach, we compared healthy and disease model organoids using the microfluidic qOBM setup. Tuberous Sclerosis Complex is a developmental disorder that affects multiple systems causing nonmalignant hamartomas that can affect the skin, heart, kidney, lung, and brain.^39^ In the brain, TSC is associated with epilepsy, autism & intellectual disability and is characterized by loss-of-function mutations in the *TSC1* and/or *TSC2* genes.^2, 40^ These proteins regulate the mammalian target of Rapamycin (mTOR) pathway.^41^ Loss of *TSC1/TSC2* results in overactivation of the mTOR pathway, which is implicated in numerous biological processes related to cell growth, proliferation, metabolism, and protein synthesis.^2^ Previous work in developing *in vitro* neuronal stem cell models of TSC have increased our understanding of the disease expression. 2D and 3D *in vitro* cultures revealed minor differences in phenotypic expression between TSC and healthy controls during early neuronal differentiation.^2, 42–44^ These differences included increased neural rosette sizes, increased neural progenitor (NPC) proliferation, increased cell size, and increased cell death. However, during later stages of neuronal differentiation, noticeable differences in neuronal and astroglial differentiation were observed, with a preference for the astro-glial fate over neuronal fate observed in cells with TSC mutations.

**Figure 3.**
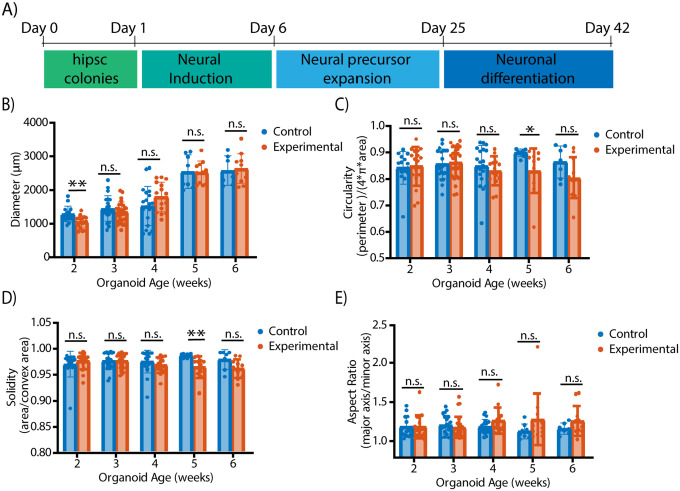
Whole-organoid morphology brightfield analysis of organoids with TSC mutations and healthy controls. (A) Schematic of protocol for TSC organoid culture. (B-D). Quantification of brightfield metrics related to organoid shape and size of the control organoids (left) and the experimental organoids (right); aspect ratio (B), circularity (C), diameter (D), and solidity (E). Images were taken using the microfluidic device. N = 15 organoids (control) and N= 17 organoids (experimental) for Week 2, N = 21 organoids (control), N = 27 organoids (experimental) for Week 3, N = 19 organoids (control), N = 15 organoids (experimental) for Week 4, N = 8 organoids (control), N = 11 organoids (experimental) for Week 5, N= 7 organoids (control), N = 11 organoids (experimental) for Week 6. Using a two-tailed unpaired t-test with Welch-correction for the 2 groups. Data is representative of 4 independent experiments with 6–8 organoids from each experiment group per experiment. Due to limited sample availability, data were pooled from both microfluidic and conventional cultures. Week 1 - Week 4 data: combination of microfluidic and conventional culture. Week 4- Week 5 data: microfluidic culture only. Organoids with low quality brightfield images were discarded from the analysis.

We hypothesized that longitudinal non-invasive imaging could help identify subtle morphological changes in organoid phenotypes. The protocol for TSC organoid culture was performed according to previously described methods for cortical spheroid formation (Figure 3A).^5, 11, 45^ In this study, we performed multiple rounds of imaging where we imaged organoids for 6 weeks of the culture process during the neural precursor expansion and neuronal differentiation processes. Using our high-content label-free imaging approach, we characterized organoid development via various metrics ranging from the whole organoid morphology to changes in neural rosette size and shape to more subtle changes in cell content over the course of the differentiation. These metrics were obtained from patient-derived organoids and used to identify differences between organoids with TSC mutations and healthy controls. Organoids with TSC mutations are considered our experimental group and were obtained from 3 different cell lines (TSC01, TSC10, TSC10E2) and our healthy controls were obtained from 2 different cell lines (PGP1, C1–2).

### 1.4 Analysis of whole organoid morphology

Using metrics obtained from brightfield imaging and qOBM imaging, we measured morphological changes that occur during organoid development on the whole-organoid. We obtained various brightfield metrics describing organoid size and shape (Figure 3B–E). Using the diameter as a measure of organoid growth, we observed that although the initial population of TSC-experimental group organoids appears smaller than the healthy controls, the diameters of organoids from both groups become comparable over time. We also note that the organoid diameters increase over time, as expected. Next, we measured the circularity, solidity, and aspect ratio to characterize the organoid shape. While none of these variables demonstrate consistent statistically significant differences in the mean between the control and experimental organoids, we see the early onset of trends indicative of the differences in organoid formation seen in previously studied day 40+ organoids with TSC mutations.^2, 42, 43^ For instance, the circularity of the TSC-experimental group organoids appears to slightly decrease over time. In contrast, the circularity of the control organoids appears more constant throughout the experiment. Similarly, the solidity of the TSC-experimental organoids also decreased over time, while the solidity of the control organoids appeared constant. Additionally, organoids with TSC mutations had fairly consistent aspect ratios over development time; however they demonstrated increased variation compared to the control organoids with significant f-tests in week 4 (F_(18,14_)=0.34, p=0.015), week 5 (F_(7,10_)=0.09, p=0.003), and week 6 (F_(6,10_)=0.16, p=0.027). This variation indicates the formation of more complex structures in the TSC. Interestingly, at day 25 (midway through week 3 of culture), the neural medium supplied to the organoids is supplemented with media to promote the differentiation of neural progenitors. Thus, the results suggest that the changes observed may be due to differences in neuronal progenitor cell proliferation or potential differentiation.

**Figure 4.**
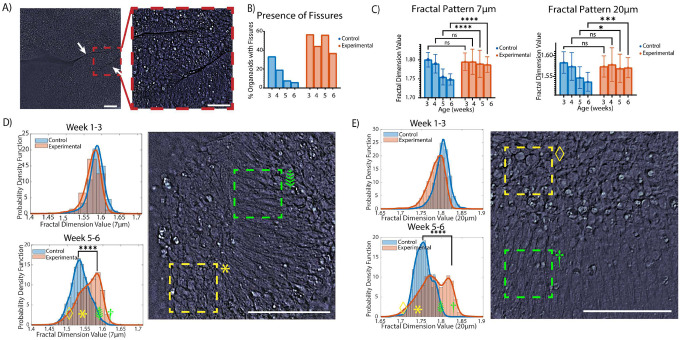
Low magnification (20X) qOBM imaging of the two experimental groups reveals differences in cell morphologies on the surface of the organoid. (A) Representative qOBM images showing several folds within experimental group organoids and control organoids at day 15 (week 2). Scale bar: 200 μm. (B) A representation of how the folds present in day 30 organoids (week 4). Right image shows a zoomed-in region with directional cells around the folding lines. (C) shows the presence of fissures over time, with the control organoids showing a decrease in fissures throughout development. Experimental group organoids do not show the same level of decrease. (D) represents the values of the fractal features for the 7 μm and 20 μm patterns corresponding to the elongated cells along the fissures. These values follow the same trend in the controls and experimental organoids as exhibited in (C). Significant differences exist between the control and experimental group organoids post- exposure to neuronal differentiation media. (E) shows the distribution of fractal values before and after culture in neuronal differentiation media to demonstrate the decrease of directional cells among the control group. They also contain images to show how different fractal values appear within the distribution. The selected images are border regions that contain both directional and circular cells. Significance was calculated using a two-tailed unpaired t-test with Welch-correction for two groups. All scale bars are 100μm.

To get a detailed analysis of the trends observed with brightfield imaging, we performed a similar analysis with qOBM to observe structural changes occurring on the organoid surface, up to 190μm into the organoid. Low magnification imaging (20X) with qOBM revealed that the TSC-experimental group organoids had more folds and complex structures forming on their surface when compared to the control (Figure 4A) with markedly improved distinction between the control and experimental groups at later time points compared to the brightfield metrics. Specifically, the qOBM images revealed internal organoid fissure lines where an organoid appeared to have folded up on itself. An example of the fissure lines can be seen in Figure 4A. Both control and TSC-experimental group organoids exhibit these folding structures; however, the number of organoids with observable folds were lower for the control group, even at early time points, and decreased rapidly over time. In contrast, a larger percentage of TSC-experimental group organoids exhibited fissures and folds, and the percentage did not decrease over time, as seen in Figure 4B. At the fissure lines, the cells along the edge appeared as directional, elongated, well-aligned cells not seen elsewhere in the organoid. Areas with (green box) and without (yellow box) directional cells can be seen in Figure 4D&E.

### 1.5 Analysis of cellular morphologies

Automated feature analysis was conducted to analyze differences in image texture and shape of organoids that presented fissure lines on their surface. We found trends in fractal dimension that correlated with the areas of the organoid containing directional cells. In particular, curved patterns corresponding to the cellular membrane and boundary were identified. Specifically, fractal dimension at scales of 5–9 μm and 17–23 μm were identified as corresponding to the oblong shape of directional cells – we selected the middle fractal values from those ranges (7 μm and 20 μm) for comparative analysis. We tracked the presence of features over time (Figure 4D&E), comparing the TSC-experimental group organoids to the controls, and found that the prevalence of these features displays a similar pattern as seen in the presence of fissures in the organoids (Figure 4C) where the fractal dimension values decreased over time in the controls but remained constant in the TSC-experimental organoids with significant differences (p<=0.01) after Week 4, which is post-exposure to neuronal differentiation medium. The fractal values plotted indicate that fewer of the elongated, directional cells surrounding the folding and edges of the organoids are present in the control group post-exposure to neuronal differentiation medium. We further analyze those fractal values distribution in images pre-and-post-neuronal differentiation medium (histograms in Figure 4D&E). Pre-exposure to neuronal differentiation medium (Week 1–3), the distributions of the TSC-experimental and control organoids overlap and display no significant differences. This suggests insignificant differences in the number of elongated, directional cells imaged. Post-exposure to neuronal differentiation medium (Week 5–6), the control distribution shifts left, i.e., displaying fewer elongated, directional cells. The TSC-experimental distribution post-exposure to neuronal differentiation medium also exhibits a skew towards fewer directional cells, albeit more subtle. However, more importantly, these organoids may maintain more cells containing directionality, as seen in Figure 4D&E. This distribution suggests that both the control and TSC-experimental organoids start with a similar number of areas that exhibit these elongated, directional cell phenotypes, but post-exposure to neuronal differentiation medium, the controls do not show this phenotype. At the same time, it persists significantly in the TSC-experimental organoids.

### 1.6 Analysis of rosette morphology

**Figure 5.**
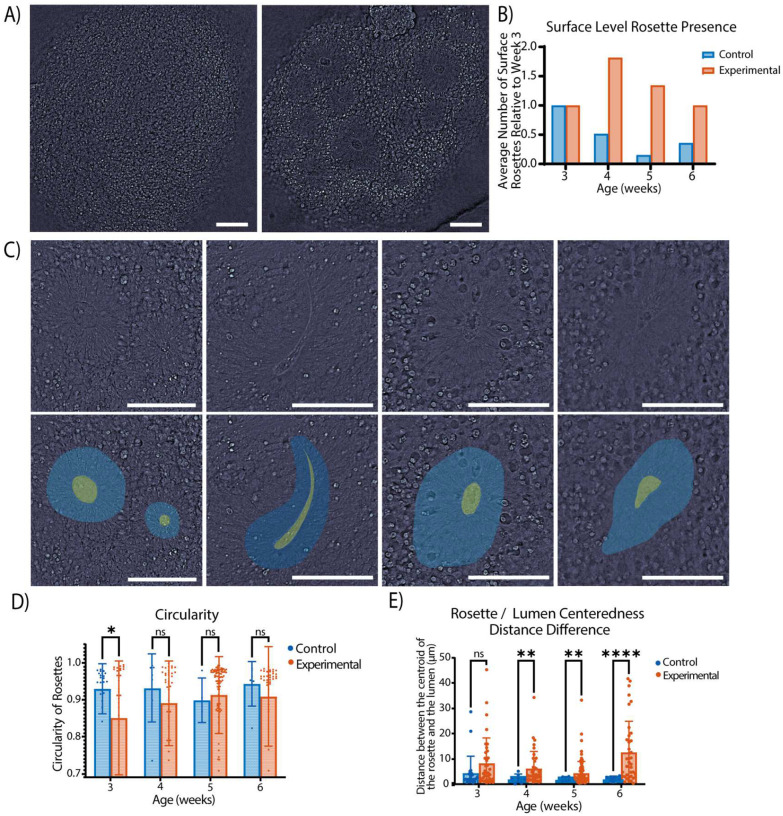
A demonstration of rosettes in the organoid. (A) (left) shows a control organoid with no surface-level rosettes and (right) shows a TSC organoid with 10 rosettes visible in the field of view. (B) shows that the TSC-experimental organoids demonstrated a statistically significantly higher number of rosettes on the surface of the organoid at all time points after Week 3. (C) shows the segmentation of 4 different rosettes. From left to right, they show a pair of rounded rosettes with the lumen centered, an irregularly shaped rosette with a centered lumen, a rounded rosette with a lumen not centered, and an irregularly shaped rosette with an uncentered lumen. (D) shows the circularity of rosettes over time. Note the larger variance in the TSC rosette circularity compared to the controls. (E) shows the distance between the center of the rosettes and the center of the lumen. Note how the lumen is less centered in the TSC-experimental rosettes than in the controls and how those differences increase over time. Significance was calculated using a two-tailed unpaired t-test with Welch-correction for two groups. All scale bars are 100 μm.

Next, we studied the differences in the number of rosettes and their morphology in control and TSC-experimental organoids. The rosettes of the organoids are lumen-filled structures with a cellular arrangement of elongated processes that radiate outward from a central core. Prior studies have not revealed differences in the number of rosettes at early time points of TSC-experimental organoid culture.^42, 46^ However, when scanning through the surface layers of the organoids, significantly more rosettes were observed in the TSC-experimental organoids versus the controls. qOBM imaging consists of surface-level imaging of approximately 190 μm into the organoid, so while we cannot make claims regarding rosette count or development in the entire organoids, we observe that the TSC-experimental organoids contain a greater number of rosettes growing close to the organoid surface and outer layers. One such representation of the increased number of surface-level rosettes can be seen in Figure 5A, where Week 4 organoids are compared: the control organoid(left) shows no rosettes, while the TSC- experimental (right) exhibits a large number of rosettes on a single plane. Figure 5B compares rosette count between TSC-experimental and control organoids. We note that the TSC-experimental organoids exhibit relatively constant surface-level rosettes over time. In contrast, the control organoids show decreased rosettes growing near the surface.

We also observed that neural rosettes in TSC-experimental organoids tended to be more densely packed and irregularly shaped than those in the control organoids. Hence, we analyzed the rosettes’ size, shape, morphology, and image texture. We calculated the circularity of the rosette and lumen and the centeredness of the lumen in the rosette. Examples of centered, non-centered, rounded, and irregularly shaped rosettes can be seen in Figure 5C. We found that the lumens of the rosettes were significantly less centered in the rosettes of the TSC-experimental organoids, as seen in Figure 5E. We also noted a disparity in the rosette circularity of the TSC-experimental groups versus the control organoids post-exposure to neuronal differentiation medium, as seen in Figure 5D. We found significant statistical test scores (f and t) between the control and TSC-experimental organoids post-neuronal differentiation, indicating a greater variation in the TSC-experimental rosette population and their mean. These findings agree with previous *in vitro* studies where the TSC-experimental organoids were observed to have altered neural rosette morphologies, possibly due to mTOR overactivation in neural progenitor cells.^44, 47^

### 1.7 Analysis of cellular content

**Figure 6.**
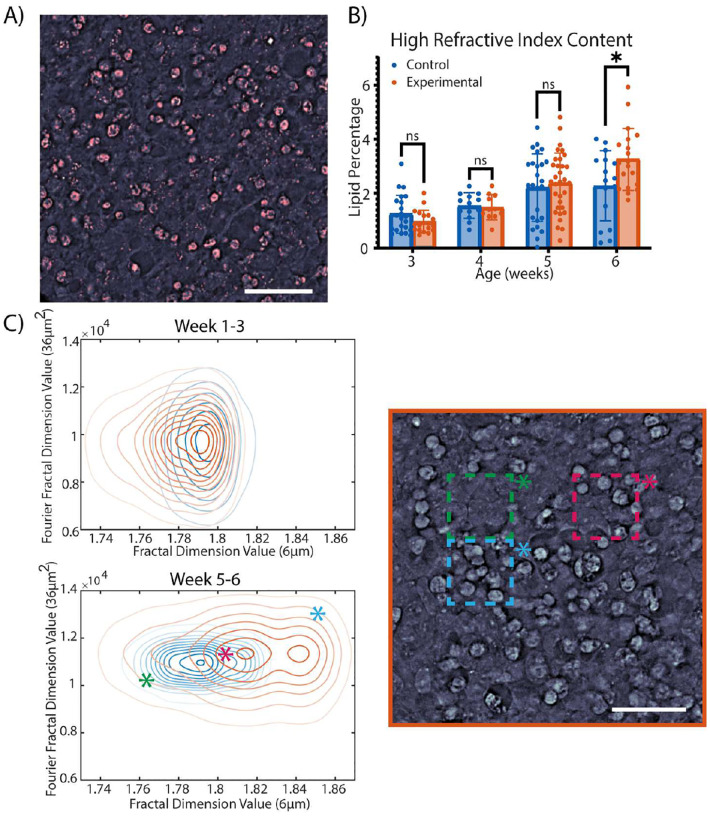
Analysis of cell content using refractive index information. (A) shows qOBM segmentation with pink as the segmented high RI material. (B) shows the lipid data shows the percentage of the organoid composed of lipids. We note the growth over time with significant differences between the control and TSC organoids in Week 6 of organoid culture. (C) shows the distribution of fractal values with higher values representing repeated pattern values. The heat maps show the distribution of the linear fractal pattern (x-axis) and the 2D circular fractal pattern (y-axis) pre-and-post-exposure to neuronal differentiation medium. The image on the right exhibits sample regions and the corresponding fractal values in an experimental organoid growing in neuronal differentiation medium. Significance was calculated using a two-tailed unpaired t-test with Welch-correction for two groups. All scale bars are 50μm

During imaging we noted that more high-refractive index, bright spots appeared in the TSC-experimental organoids than in the control organoids. In qOBM imaging, a brighter region corresponds to an area with a higher refractive index (RI), implying the presence of lipid droplets, cellular membranes, and nucleic material. The high RI material was segmented from the image using a Δ*n>*=1.46 (Figure 6A). Figure 6B shows that as the cultures grew, the control and TSC-experimental organoids increased in high RI content. However, the TSC-experimental organoids increased at a greater rate. By the final week of the study, the difference between the TSC-experimental and control organoids was statistically significant (p=0.02).

Leveraging the quantitative nature of qOBM imaging, we used automated feature analysis to compare the amount of high RI spots between the organoid types. We identified the fractal values corresponding with the high RI droplets, including a curved linear fractal dimensions with a curvature diameter between 4–8 μm and a two-dimensional circle fractal pattern corresponding to circular shapes of 34–38 μm^2^. These fractal patterns show significant differences (p<=0.01) between the TSC-experimental group and control organoids post-exposure to neuronal differentiation medium indicating differences in the presence of the high-RI material, as seen in Figure 6C. In further exploring the distributions of the fractal values, we see that prior to neuronal differentiation, the control and TSC-experimental group organoids share a similar distribution, with many of the values overlapping (as seen in Figure 6C). Post-exposure to neuronal differentiation medium, a shift occurs in which the control and TSC-experimental group organoids exhibit higher overall fractal values (indicating greater fractal structures than pre-exposure to neuronal differentiation medium and greater high-refractive index content). Post-exposure to neuronal differentiation medium, we also observe that the TSC-experimental organoids exhibit larger fractal values than the control organoids with a larger distribution, indicating higher heterogeneity of cellular structures. As indicated by the stars overlaid on the heat maps, even a tiny subsection of the organoid can have vastly different fractal values based on the structures in the sections analyzed. We note that the control organoids have a smaller, more homogeneous distribution of values post-exposure to neuronal differentiation medium; meanwhile, the TSC-experimental organoids display a larger overall distribution with higher value nodes, as seen in Figure 6C. These results suggest that organoids display areas of varying lipid concentration, with some areas possessing a higher percentage than others. We hypothesize that these spatial differences observed might be due to the distribution of different cell types in these areas.

### 1.8 Lipid staining and histology verification

**Figure 7.**
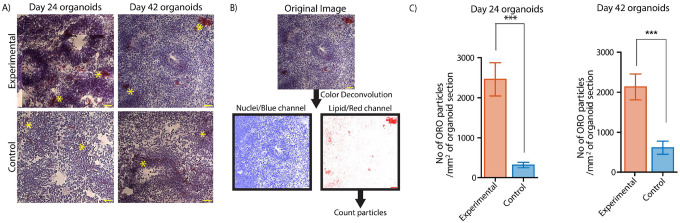
Histological assessment reveals similar cell morphologies and lipid content differences in experimental and control organoids to non-invasive imaging. (A) Top: Representative Oil Red O images of organoid sections showing lipid droplets (red, indicted by yellow asterisks) at day 24 for experimental (1st column) and control (2nd column) samples. Bottom: Representative Oil Red O images of organoid sections showing lipid droplets (red, indicated by yellow asterisks) at day 42 for experimental (1st column) and control (2nd column) samples. Slices were counterstained with Hematoxylin. The brightness and contrast of images were adjusted for visualization. Scale bar:40 μm. (B) Schematic showing the image processing pipeline for quantifying ORO particles in the organoid sections (C) Quantification of ORO-positive particles in organoid sections of experimental group organoids (left) and control organoids (right) at two different time points: day 24 and day 42. Using a two-tailed unpaired t-test with Welch-correction for 2 groups. D24: 112 images from 17 sections (control) and 75 images from 20 sections (experimental). D42: 81 images from 25 sections (control) and 71 from 22 sections (experimental).

To validate some of the findings from our study we performed Oil Red O (ORO) and Hematoxylin staining on organoid sections Figure 7A). Hematoxylin staining was performed to detect the cell nucleic content. Using the results from the Hematoxylin staining, we qualitatively assessed neural rosette morphology, distribution, and cell density in the organoid samples. Additionally, we performed ORO staining to identify possible reasons for differences in refractive index content between the control and TSC-experimental groups since it enables the identification of neutral lipids and lipid droplets in tissue sections.^48^ We qualitatively observed that the TSC-experimental organoids, on average, tend to have more nucleic content than the healthy controls, as indicated by the deeper expression of the hematoxylin stain in the TSC-experimental group organoid samples. These results are consistent with previous findings on increased cell size and cell division in TSC-experimental samples compared to controls. The neural rosette structures in the TSC-experimental group organoids were also more densely packed than those in the control organoids.

To measure the lipid content of the organoid samples, we quantified the amount of ORO particles in the organoid sections (Figure 7B). Our results revealed that TSC-experimental group organoids had a higher ORO+ particle content than healthy controls both before (day 24) and after exposure to neuronal differentiation medium (day 42) (Figure 7C). These differences in lipid distribution between TSC-experimental group organoids and healthy controls could explain disease expression in these organoids, as over-accumulation of lipids in neural stem cells has been associated with deregulating their ability to proliferate and differentiate.^49^ Overall, our results highlight the utility of our non-invasive approach in identifying subtle differences in cell content and metabolism, which can be investigated further with downstream molecular assays.

## 2 Discussion

Organoids have been shown to demonstrate superior fidelity to human development compared to animal models in both typical and pathological scenarios. Nevertheless, effectively tracking these 3D systems in a non-invasive, *in situ* manner remains a challenge. This research employs a label-free, non-invasive imaging technique to continuously observe diseased organoids and provides cellular and subcellular distinctions continuously over time. The platform is also capable of capturing developmental differences beginning at previously unstudied early time points. These capabilities enable the detection of variations in neural rosette morphology, structural development, and lipid metabolism between healthy and disease model organoids.

Our neural rosette morphology results were consistent with previous findings. Some morphological changes we observed indicated changes in structure formation and lipid metabolism. For example, we observed increased folds and complex structure formation in TSC-experimental organoid models in early-stage organoids. However, we have yet to identify a potential cause for this observation. Previous brain organoid studies have however highlighted the role of ASD-related genes such as *PTEN* on cortical folding.^13^ It was discovered that the down-regulation of the *PTEN* gene in neural progenitor cells (NPCs) led to increased cortical expansion and folding in cerebral organoids.^13^ The down-regulation of *PTEN* could provide one explanation for the more complex structures observed in the TSC-experimental organoids due to its inhibitory role on *PI3K*, a known antagonist of TSC1/TSC2.^50^ However, further investigation is needed to confirm this.

We also observed increased high refractive index content, which maybe attributed to lipids, in the TSC-experimental organoids compared to their healthy controls. Our results indicate possible dysregulation of lipid metabolism in the TSC-experimental organoids via quantification of the qOBM images and ORO staining. One limitation of the ORO staining is that it is a non-specific stain.^48^ As a result, it is difficult to determine what lipid species may be affected by the TSC mutation. Additionally, ORO staining helps detect hydrophobic and neutral lipids and not polar lipids (e.g., sphingolipids), which play critical roles in cognitive; development^51–53^ hence, our findings only provide an estimate of lipid presence in the organoid sections. Further, ORO staining is an endpoint measurement. As such, the tissue used for ORO staining can not be grown further or used for additional analysis. As such, a limited sample size was used. Additionally, the qOBM segmentation procedure described in this paper does not isolate only lipids in the staining process. The segmentation also includes nucleic material and fats and lipids in the tissue since these structures have a high RI. With some limitations, the qOBM image segmentation can be used as a proxy to estimate lipid accumulation, as we cannot separate lipids from other cellular content. As such, we do not have a one-to-one agreement with the ORO staining data, but the results do show a general agreement in that the TSC-experimental organoids contain greater lipid or high RI content at later days. Further lipidomics studies are necessary to uncover the underlying mechanisms behind the observations made in this paper and identify what cell types may be contributing to the lipid count differences.

Indeed other optical methods have been used to image spheroids and organoids, including other forms of quantitative phase imaging (QPI) and dynamic full field (DFF)-OCT, however these methods have important limitations. Previously implemented phase imaging methods have mostly been restricted to thin samples (<100μm) as they operate in transmission mode, or they depend on polarization and have limited subcellular detail.^54–56^ Alternatively, DFF-OCT can provide similar structural information as qOBM with similar penetration depth,^57^ but is more complex, expensive and data-intensive as many acquisitions are necessary to render the structural detail.^57^ On the other hand, qOBM is fast, low-cost, simple, and can even render similar dynamic/metabolic activity information.^34^ Thus, qOBM offers both high throughput and high-subcellular detail in an easy-to-use and low-cost embodiment.

In this work, we demonstrated an effective tool to non-invasively monitor human brain organoids over cell culture, providing access to morphological changes occurring over time without disrupting the cellular environment. Using this microfluidicsenabled imaging method, we performed a high-content analysis which revealed differences in cell morphology and content between TSC-experimental organoids and healthy controls. The ability of qOBM to provide cellular and sub-cellular detail in 3D up to 190μm into the organoid is key to enabling unique quantitative features in the organoids. Differences in the folding of the organoids, shape of the organoids, distribution of lipids and differences that occur within the rosette structures of the organoid were highlighted in this study.

Utilizing the microfluidic platform enabled the tracking of culture and monitoring of these live organoids while minimizing the risk of contamination due to the ability to provide automated cell feeds. The device was also specifically design to enable qOBM and brightfield imaging, which jointly enable the tracking of individual organoids over time to compare intra-organoid heterogeneity, a feature that will be used in future studies. Enabled by microfluidic technology, we demonstrate that our imaging approach provides detailed high-content information about organoid development during the culture process. We envision this combined qOBM-microfluidics technology being used to quantify important structural properties of organoids to help improve our understanding of endogenous developmental processes and better guide organogenesis and disease modeling.

## 3 Methods

### 3.1 Device Fabrication for qOBM Imaging

Device fabrication was conducted using previously published protocols^37^ but with modifications. The device design was drawn in SolidWorks, and molds for the devices were made using 3D printing by the company Protolabs. The molds were printed in the material Accura SL 5530. Using the 3D printed molds, microfluidic devices were fabricated in polydimethylsiloxane (PDMS) (Dow Corning Sylgard 184, Midland, MI) by soft lithography.^58^ Briefly, PDMS was mixed in a 10:1 ratio of pre-polymer and crosslinker, degassed to remove air bubbles, poured on the master mold, degassed a second time to remove remaining bubbles, and cured overnight at 80°C. Following curing, PDMS devices were peeled off the master molds. The molds were not pre-treated prior to use. Additionally, creating the crossflow in the device required two-layer PDMS fabrication. The mold for both layers was identical. For both layers of features, PDMS was poured on the mold to a height of approximately 3 mm to define the height of the culture chamber. Following curing and peeling, cylindrical chambers were made in both feature layers by manually punching holes with an 8 mm biopsy punch (VWR). Inlet and outlet holes were punched with a 2 mm biopsy punch (VWR). The bottom PDMS layer was bonded to a 1 mm thick glass slide. Next, the top and bottom PDMS layers were plasma bonded together and left in an oven at 80°C overnight to strengthen the bond.

### 3.2 Free-Space qOBM System

The qOBM system consists of a conventional brightfield microscope with a modified illumination module.^24–26, 32^ Rather than the classic transmission-based illumination used in brightfield microscopy and QPI, qOBM illuminates samples in epi-mode using four LED light sources (720 90 nm) deployed through multimode optical fibers arranged around the objective, 90° from each other. Through these fibers, the sample is illuminated in epi-mode, where approximately 45 mW are incident on the organoid samples. In the organoids, the photons undergo multiple scattering events causing the photons to change direction, with some being redirected back toward the microscope objective. These redirected photons create an effective virtual light source within the sample with an overall oblique illumination, a process known as oblique back-illumination.^59^ Variations in the index of refraction in the sample refract the light either towards or away from the microscopy objective, resulting in intensity fluctuations that encode the RI properties of the sample. This work uses a Nikon S Plan Fluor LWD 99 20X, 0.45 NA, and Nikon S Plan Fluor LWD 40X, 0.6 NA. Light collected by the microscope is detected using an sCMOS camera (pco.edge 4.2 LT). Intensity images collected from two opposing illumination angles are subtracted to produce a differential phase contrast (DPC) image. Two orthogonal DPC images (a total of four acquisitions) are deconvolved with the system’s optical transfer function to finally obtain quantitative phase contrast images. This process has been described in further detail in previous studies.^24–26, 32, 33^

### 3.3 Organoid Culture

hiPSCs derived from patient donors were used for organoid generation. TSC-experimental organoid formation was performed using pre-established protocols for generating cortical organoids.^5, 11, 45^ Briefly, hiPSC colonies were dissociated from a layer of mouse embryonic fibroblast feeders by exposing them to a low concentration of dispase for approximately 30 min. Suspended colonies were transferred into ultra-low-attachment 100 mm plastic plates in hiPSC medium without FGF2. The medium was supplemented with the ROCK inhibitor for the first 24 h (day 0). Dorsomorphin and SB-431542 were added to the medium for the first five days for neural induction. On the sixth day in suspension, the floating spheroids were moved to neural medium (NM) containing Neurobasal, B-27 serum substitute without vitamin A, GlutaMax, 100 U ml^−1^ penicillin, and 100 μl streptomycin. The NM was supplemented with 20 ng ml^−1^ FGF2 and 20 ng ml^−1^ EGF for 19 days with medium change every other day. To promote differentiation of the neural progenitors into neurons, FGF2 and EGF were replaced with 20 ng ml^−1^ BDNF and 20 ng ml^−1^ NT3 starting on day 25 till day 42 of the culture. Organoids were cultured in the microfluidic devices starting on day 14. Prior to day 14, organoids were cultured in 6-well plates on an orbital shaker. A description of the cell lines used in this study is provided in Table 1.

The healthy control organoids were derived from patients with no TSC mutations. In contrast, the TSC01 and TSC10 organoids were derived from patients with mutations in their *TSC2* gene. The isogenic control organoid, TSC10E2, was derived from an hIPSC cell line of a healthy patient (PGP1) which was genetically modified by knocking in mutations into the *TSC2* gene via CRISPR-Cas 9 gene editing. All studies were approved by Emory University School of Medicine Institutional Review Board (IRB). All methods and experimental protocols were in accordance with institutional guidelines. All subjects or their legal representatives were informed and signed informed consents.

### 3.4 Brightfield Imaging and Quantification

Brightfield images of devices were acquired prior to the start of the qOBM imaging session using an EVOS microscope. Organoid size and shape features were quantified from images using FIJI/ImageJ. Multiple images of different regions of the same organoid had to be taken for larger organoids because the organoids were larger than the field of view on the EVOS microscope. The images were then stitched together using a pair-wise stitching plugin on FIJI/ImageJ before quantification.^60^

### 3.5 Imaging Organoids with qOBM

A total of 24 control and 24 TSC-experimental organoids were imaged over a four-week time period from day 15 of culture (week 3, pre-neuronal differentiation) to day 42 of culture (week 6, post-neuronal differentiation). The organoids were imaged inside microfluidic devices 3 times a week with two different magnification objectives: (1) a 20X (0.45NA, 850 μm^2^ fields of view (FOV)) microscope objective captured images of the organoid up to 190 μm in-depth, and (2) a 40X (0.6NA, 425m^2^ FOV) microscope objective captured additional, higher resolution images taken of features of interest, including cell structure, rosette morphology, and unique features that had not previously been well-resolved with the 20X objective. A subset of organoids were taken for endpoint imaging at day 24, and the remaining subset was processed for endpoint imaging at day 42. An air stream incubator was used during imaging to helped maintain homeostatic temperatures.

### 3.6 Manual Feature Analysis of qOBM images

Post-imaging, qualitative and quantitative features were extracted from the organoid images, including the presence (enumeration) of observed folds and rosette presence. Additionally, the rosettes and lumen were manually segmented for quantitative analysis. Finally, segmentation was used to separate the content of high-RI particulate structures, corresponding primarily to what we hypothesize to be lipid and nucleic acid material. The relationship between the measured optical phase and the sample’s refractive index is given by,

(2) $$\Delta n=\frac{\lambda \Delta \phi }{2\pi \Delta z}$$

Where Δ*n* = *n*_0_ −*n*_*m*_ represents the refractive index difference between the object (*n*_0_) and the surrounding medium (*n*_*m*_), *λ* represents the wavelength, Δ*φ* represents the phase difference, and Δ*z* represents the thickness of the object, or in this case, the effective thickness of the slice provided by this 3D imaging method.^25^ Here we take the medium’s refractive index (*n*_*m*_) to be that of a PDMS scattering medium used in imaging to simulate thick brain tissue, 1.3440.^61^ For all 20X images, a RI threshold (n>=1.46) was used to segment the lipid and nucleic acid material from the rest of the organoid cells in the images.^26, 62^

### 3.7 Automated Feature Analysis of qOBM images

Quantitative image features extracted from the qOBM images were analyzed to assess structural differences between the control and TSC-experimental organoids. To accomplish this, images from the 20X, 0.45 NA objective (720μm × 720μm) were subdivided into regions of 50μm × 50μm. With those subdivided regions, features per region were extracted based on texture analysis,^63^ fractal analysis,^64^ Fourier space features,^65^ and mathematical auto-correlation transformations.^66, 67^ Feature selection ranking was performed using Minimum Redundance and Maximum Relevance, Neighborhood Component Analysis, and the Chi-square tests, as implemented by Matlab’s functions fscmrmr, fscnca, and fscchi2, respectively. The highest 150 ranked features from all three methods that posed the greatest differences between the data sets are discussed in the Results.

### 3.8 Oil Red O staining and Quantification

The Oil Red O staining protocol was provided by Aqua Asberry, the Laboratory Coordinator for the Parker H. Petit Institute for Bioengineering and Bioscience Research Histology Core at the Georgia Institute of Technology in Atlanta, GA. Briefly, the frozen sections were allowed to thaw and air dry at room temperature for about 10 mins. Next, the sections were submerged in Oil Red O staining reagent for about 20 mins. Following ORO staining, the samples were washed under running water while being careful to avoid direct contact with the water. A counterstain with Hematoxylin was also performed for about 20 secs, and the samples were rewashed under running water until all the excess stains had been eliminated from the washing solution. The samples were then mounted on a coverslip using an aqueous mounting medium. Samples were then imaged using a Zeiss AxioObserver Z1 Fluorescent Microscope. Quantification of total ORO lipid content was performed using Fiji/ImageJ software. Color deconvolution separated the red channel (ORO staining) from the blue channel (Hematoxylin staining) using the built-in matrix: FastRed/FastBlue. Following color deconvolution, the images were thresholded, and the number of pixels was counted to determine the number of ORO particles in the image. This number was normalized by the image area occupied by the pixels.

## Figures and Tables

**Table 1.** Summary of hiPSC lines used in qOBM study

Name of Cell Line	Description
C1-2	Healthy Control
426	Healthy Control
PGP1	Healthy Control
TSC10E2	Isogenic Control
TSC10	Patient Sample
TSC01	Patient Sample

## Data Availability

The data that support the findings of this study are available from the corresponding author upon reasonable request.
